# Mortality After Exposure to a Hurricane Among Older Adults Living With Dementia

**DOI:** 10.1001/jamanetworkopen.2023.2043

**Published:** 2023-03-07

**Authors:** Sue Anne Bell, Marie Lynn Miranda, Julie P. W. Bynum, Matthew A. Davis

**Affiliations:** 1 Department of Systems, Populations, and Leadership, University of Michigan School of Nursing, Ann Arbor; 2 Children’s Environmental Health Initiative, University of Notre Dame, Notre Dame, Indiana; 3 Department of Internal Medicine, University of Michigan Medical School, Ann Arbor

## Abstract

This cohort study examines mortality after hurricane exposure in older adults living with Alzheimer disease and other related dementias.

## Introduction

Older adults living with Alzheimer disease and other related dementias (ADRD) are especially vulnerable during disaster events because of their dependence on others during crises. Previous hurricane studies^1,2,3^ have found general increases in mortality after exposure. However, little is known about how mortality after hurricane exposure differs among older adults living with ADRD. Therefore, we examined mortality changes among older adults with ADRD exposed to major US hurricanes.

## Methods

This retrospective cohort study used Medicare administrative claims data from fee-for-service beneficiaries 65 years or older affected by Hurricanes Harvey (2017), Irma (2017), and Florence (2018). Using date of death among decedents, we calculated monthly and annual all-cause mortality among older adults with vs without ADRD. Annual mortality rates before vs after the hurricane made landfall were compared to estimate differences in risk. Attributable risks, attributable fraction, and relative risks (RRs) were calculated. Subanalyses were conducted by sociodemographic characteristics, including age category, sex, race, dual eligibility status, and number of comorbidities identified using the Elixhauser comorbidity index.^4^ Race, ethnicity, and ADRD diagnosis were identified using validated algorithms. Race and ethnicity data were collected to assess whether mortality differed among these groups. Analyses were repeated among beneficiaries in counties that did not experience a hurricane and to account for outmigration by including those who left affected areas in the year after the hurricane. Informed consent was waived because as a secondary data analysis the research involved no more than minimal risk to participants. The study received institutional review board approval from the University of Michigan and followed STROBE reporting guideline.

## Results

The study population consisted of 346 171 beneficiaries who resided in 139 hurricane-affected counties with a Federal Emergency Management Agency disaster declaration^5^ compared with 352 616 beneficiaries in 437 counties in the same state that did not have a disaster declaration. In the year after hurricane exposure, 54 340 older adults with ADRD died (mean [SD] age, 85.4 [8.2] years; 32 426 women [59.7%] and 21 914 men [40.3%]; 52 631 [96.9%] White, 5562 [10.2%] Black, and 6157 [11.3%] other race and ethnicity, including American Indian or Alaska Native, Asian or Pacific Islander, Hispanic, other, and unknown). Mortality peaked 3 to 6 months after Hurricanes Irma and Harvey landfall (Figure) but not after Hurricane Florence. Overall, 1.32 deaths (95% CI, 1.13-1.52 deaths) per 1000 population were attributed to hurricane exposure among the ADRD population (RR, 1.08; 95% CI, 1.07-1.09) (Table). Risks were lower in counties that did not experience a hurricane (attributable risk, 0.63; 95% CI, 0.42-0.84; RR, 1.03; 95% CI, 1.02-1.05). Mortality attributed to exposure among the ADRD population ranged from 10.9% for Hurricane Harvey to 6.2% for Hurricane Irma. Mortality risk was highest among ADRD subgroups 85 years or older (RR, 1.09; 95% CI, 1.08-1.11) and the dually eligible (RR, 1.11; 95% CI, 1.07-1.12). Examination of outmigration, by including all residents in affected counties, showed that the magnitude of the association persisted (RR, 1.09; 95% CI, 1.08-1.10).

**Figure.**
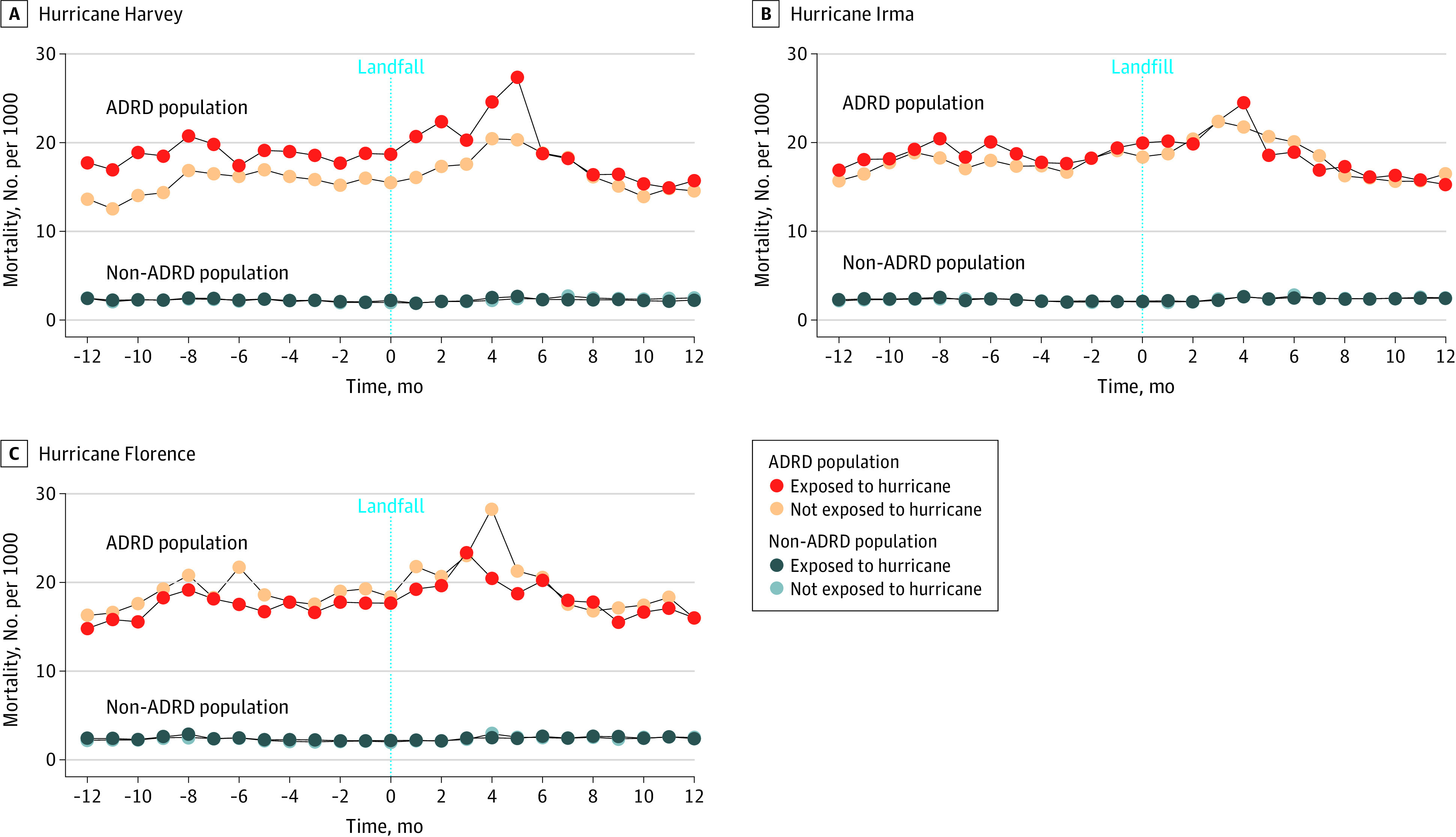
All-Cause Mortality Associated With Exposure to Hurricanes Among Older Adults With Alzheimer Disease and Other Related Dementias (ADRD) vs Non-ADRD Populations

**Table.** Mortality Among Older Adults Living With Dementia After Hurricane Exposure by Characteristic

Characteristic	No. (%) of ADRD deaths year after hurricane	Morality rate, No. of deaths per 1000		Attributable risk, No. per 1000 population (95% CI)	Attributable fraction, % (95% CI)	Relative risk (95% CI)
		Year before	Year after			
**Mortality among older adults living with dementia in counties with a FEMA disaster declaration**						
Overall	54 340	16.7	18.1	1.32 (1.13 to 1.52)	7.3 (6.2 to 8.3)	1.08 (1.07 to 1.09)
By hurricane						
Harvey	9856 (18.1)	16.3	18.3	2.00 (1.54 to 2.46)	10.9 (8.5 to 13.3)	1.12 (1.09 to 1.15)
Irma	36 135 (66.5)	16.8	17.9	1.10 (0.87 to 1.34)	6.2 (4.9 to 7.5)	1.07 (1.05 to 1.08)
Florence	8349 (15.4)	17.2	18.7	1.50 (0.98 to 2.01)	8.0 (5.3 to 10.6)	1.09 (1.06 to 1.12)
By characteristic						
Age category, y						
65-74	5829 (10.7)	10.5	10.8	0.29 (−0.07 to 0.64)	2.7 (−0.6 to 5.9)	1.03 (0.99 to 1.06)
75-84	15 820 (29.1)	11.3	11.9	0.62 (0.38 to 0.86)	5.2 (3.2 to 7.2)	1.05 (1.03 to 1.08)
≥85	32 691 (60.2)	22.4	24.5	2.12 (1.78 to 2.47)	8.7 (7.3 to 10.0)	1.09 (1.08 to 1.11)
Sex						
Men	21 914 (40.3)	18.5	19.8	1.26 (0.93 to 1.60)	6.4 (4.7 to 8.0)	1.07 (1.05 to 1.09)
Women	32 426 (59.7)	15.7	17.1	1.39 (1.15 to 1.63)	8.2 (6.8 to 9.5)	1.09 (1.07 to 1.11)
Race						
Black	5562 (10.2)	17	17.8	0.86 (0.25 to 1.46)	4.8 (1.4 to 8.1)	1.05 (1.01 to 1.09)
White	42 631 (96.9)	17.2	18.8	1.56 (1.33 to 1.79)	8.3 (7.1 to 9.4)	1.09 (1.08 to 1.10)
Other^a^	6147 (11.3)	13.7	14.3	0.56 (0.09 to 1.03)	3.9 (0.6 to 7.1)	1.04 (1.01 to 1.08)
Dual eligibility status						
Dual eligible	17 873 (32.9)	19.8	21.6	1.86 (1.45 to 2.26)	8.6 (6.8 to 10.4)	1.09 (1.07 to 1.12)
Medicare only	36 467 (67.1)	15.5	16.7	1.19 (0.97 to 1.41)	7.1 (5.8 to 8.4)	1.08 (1.06 to 1.09)
No. of comorbidities						
0-1	20 807 (38.3)	9.3	10.7	1.36 (1.17 to 1.55)	12.7 (11.1 to 14.4)	1.15 (1.12 to 1.17)
≥2	33 533 (61.7)	28.0	31.6	3.59 (3.16 to 4.01)	11.3 (10.1 to 12.6)	1.13 (1.11 to 1.14)
**Mortality among older adults living with dementia in counties in the same state without a FEMA disaster declaration**						
Overall	55 644	18.2	18.9	0.63 (0.42 to 0.84)	3.3 (2.3 to 4.4)	1.03 (1.02 to 1.05)
By hurricane						
Harvey	18 096 (32.5)	16.7	17.9	1.14 (0.80 to 1.48)	6.4 (4.5 to 8.2)	1.07 (1.05 to 1.09)
Irma	23 142 (41.6)	18.3	18.6	0.25 (−0.07 to 0.56)	1.3 (−0.4 to 3.0)	1.01 (1.00 to 1.03)
Florence	14 406 (25.9)	19.1	19.5	0.36 (0.16 to 0.55)	1.8 (0.8 to 2.8)	1.02 (1.01 to 1.03)
By characteristic						
Age category, y						
65-74	6542 (11.8)	11.7	11.6	−0.08 (−0.45 to 0.28)	0.7 (−2.5 to 3.8)	0.99 (0.96 to 1.03)
75-84	17 619 (31.7)	15.1	15.2	0.04 (−0.26 to 0.34)	0.2 (−1.8 to 2.2)	1.00 (0.98 to 1.02)
≥85	31 483 (56.6)	24.5	25.7	1.22 (0.85 to 1.60)	4.8 (3.3 to 6.2)	1.05 (1.03 to 1.07)
Sex						
Men	21 614 (38.8)	19.9	20.7	0.83 (0.46 to 1.19)	4.0 (2.3 to 5.7)	1.04 (1.02 to 1.06)
Women	34 030 (61.2)	17.3	17.8	0.56 (0.31 to 0.81)	3.1 (1.7 to 4.5)	1.03 (1.02 to 1.05)
Race						
Black	5558 (10.0)	18.7	18.1	−0.64 (−1.28 to 0.00)	3.4 (−0.0 to 6.7)	0.97 (0.93 to 1.00)
White	43 845 (78.8)	18.8	19.8	1.05 (1.04 to 1.07)	5.1 (3.9 to 6.3)	1.05 (1.04 to 1.07)
Other^a^	6241 (11.2)	14.9	14.7	−0.22 (−0.72 to 0.27)	1.5 (−1.9 to 4.7)	0.99 (0.95 to 1.02)
Dual eligibility status						
Dual eligible	19 073 (34.3)	21.1	23.2	2.10 (1.67 to 2.53)	9.0 (7.3 to 10.8)	1.10 (1.08 to 1.12)
Medicare only	36 571 (65.7)	17	17.2	0.14 (−0.10 to 0.37)	0.8 (−0.6 to 2.2)	1.01 (0.99 to 1.02)
No. of comorbidities						
0-1	23 215 (41.7)	12.3	11.8	−0.47 (−0.70 to −0.24)	3.8 (2.0 to 5.6)	0.96 (0.94 to 0.99)
≥2	32 429 (58.3)	22.9	33.1	10.16 (9.75 to 10.57)	30.7 (29.7 to 31.7)	1.44 (1.42 to 1.46)

Abbreviations: ADRD, Alzheimer disease and other related dementias; FEMA, Federal Emergency Management Agency.

^a^ Other includes American Indian or Alaska Native, Asian or Pacific Islander, Hispanic, other, and unknown.

## Discussion

Hurricane exposure was associated with increased mortality among the ADRD population after 2 of the 3 hurricanes. Mortality risks were lower among beneficiaries in counties that did not experience a disaster. Mortality peaked 3 to 6 months after Hurricanes Irma and Harvey, suggesting the increase in mortality was due to factors other than the immediate harms of the storm. The higher mortality risks associated with Hurricane Harvey are consistent with the larger scope, scale, and impact of this storm. As anticipated, mortality risk was highest among more vulnerable ADRD subgroups, including the oldest individuals and those dually eligible. This study is limited by its observational design and not designed to make causal inferences. As climate change impacts advance,^6^ an integrated approach designed to anticipate and respond to the needs of older Americans living with ADRD during disasters is critical. Improved response processes informed by research, policy, funding, and operational considerations to support the ADRD population affected by disasters are needed.

## Notes

Supplement.
Data Sharing Statement
