# Supplementary material for: Mortality After Exposure to a Hurricane Among Older Adults Living With Dementia
Source: JAMA Netw Open. 2023 Mar 7;6(3):e232043. doi: 10.1001/jamanetworkopen.2023.2043 (PMC9993175; doi:10.1001/jamanetworkopen.2023.2043)
Supplement: Supplement. — Data Sharing Statement [file jamanetwopen-e232043-s001.pdf]

Data Sharing Statement

Bell. Mortality After Exposure to a Hurricane Among Older Adults Living With Dementia. *JAMA Netw Open*. Published March 07, 2023. doi:10.1001/jamanetworkopen.2023.2043

Data
Data available: No

Additional Information
Explanation for why data not available: CMS data subjected to DUA agreement
